# *Tempo Rubato *: Animacy Speeds Up Time in the Brain

**DOI:** 10.1371/journal.pone.0015638

**Published:** 2010-12-29

**Authors:** Mauro Carrozzo, Alessandro Moscatelli, Francesco Lacquaniti

**Affiliations:** 1 Institute of Neuroscience, National Research Council, Rome, Italy; 2 Laboratory of Neuromotor Physiology, Santa Lucia Foundation, Rome, Italy; 3 Centre of Space BioMedicine, University of Rome Tor Vergata, Rome, Italy; 4 Department of Neuroscience, University of Rome Tor Vergata, Rome, Italy; Royal Holloway, University of London, United Kingdom

## Abstract

**Background:**

How do we estimate time when watching an action? The idea that events are timed by a centralized clock has recently been called into question in favour of distributed, specialized mechanisms. Here we provide evidence for a critical specialization: animate and inanimate events are separately timed by humans.

**Methodology/Principal Findings:**

In different experiments, observers were asked to intercept a moving target or to discriminate the duration of a stationary flash while viewing different scenes. Time estimates were systematically shorter in the sessions involving human characters moving in the scene than in those involving inanimate moving characters. Remarkably, the animate/inanimate context also affected randomly intermingled trials which always depicted the same still character.

**Conclusions/Significance:**

The existence of distinct time bases for animate and inanimate events might be related to the partial segregation of the neural networks processing these two categories of objects, and could enhance our ability to predict critically timed actions.

## Introduction

Timing visual events over the scale of tens to hundreds of milliseconds is essential for successful interactions with people or objects in everyday life. However the brain mechanisms involved in such time estimates are still incompletely understood. Generally, time cannot be directly measured at a given moment but requires internally generated and/or externally triggered signals over the interval to be estimated [1]–[7]. The classical view that events are timed by a centralized supra-modal clock recently has been called into question in favour of distributed, specialized mechanisms. Thus, it has been shown that the apparent duration of a dynamic stimulus can be reduced in a local region of visual space following motion adaptation [8], and the effect of this adaptation is spatially selective in real-world rather than retinal coordinates, allowing to separately time targets placed in different locations of external space [9]. In particular, local adaptation of the visual field is induced by high-temporal-frequency stimuli but not by low-temporal-frequency stimuli [8]. Apparent duration depends on several additional factors which are specific to the stimulus or the context; for instance, it depends on the stimulus visibility [10], speed [11], temporal frequency [12], predictability [13], as well as the level of attention [14], the intention to perform an action [15], or saccadic eye movements [16].

Here we consider the possibility that the neural time mechanisms also involve high-level systems differentially tuned to animate and inanimate motion. The animate-inanimate distinction is considered a foundational one, because it arises early in infancy, is cross-culturally uniform, and is critical for causal interpretations of actions and events [17]–[18]. The animate-inanimate or living-nonliving distinction hinges on expected kinetic differences. Animate entities are endowed with internal energy sources which allow self-propelled motion [17], [18]. By contrast, inanimate entities are driven by external energy sources only and are incapable of self-propelled motion. In particular, people expectations from daily life regarding how human beings move in the environment differ considerably from expectations regarding the motion of inanimate objects [19]. Indeed, there is ample evidence that the neural substrates associated with human motion processing are at least in part distinct from those associated with inanimate motion [19], [20].

Despite the wealth of psychophysical and physiological studies that addressed human and inanimate motion processing, to our knowledge the possibility that these two motion categories may exert differential top-down influences on the neural mechanisms computing time has not been tested so far. However, it has recently been hypothesized that the brain constantly calibrates its time estimation by comparing the predictions of a forward model about future positions of the moving object with the visual feedback [4], [8], [21]. If one takes this hypothesis one step further, one may surmise that the time base used by the brain to process visual motion is calibrated against the specific predictions regarding the motion of animate or inanimate figures. Because these predictions are subserved by distinct brain processes and presumably engage different forward models [21]–[23], one might expect that also the mechanisms of time calibration are distinct for animate versus inanimate events. For instance, functional magnetic resonance imaging (fMRI) revealed that activation in the posterior superior temporal sulcus and gyrus (pSTS/pSTG) increases in relation to the degree of animacy [22].

The benefit of utilizing specific calibration mechanisms according to the nature of the events being monitored may be greatest while viewing dynamic scenes and during active exploration of the environment. In such cases, keeping track of positions and motions is difficult, and potential gains from applying specialized rather than general-purpose spatio-temporal filters would be maximal. Specialization of the neural time estimates could enhance the temporal resolution of visual processing for different categories of items, and could enhance the ability to predict critically timed events. With regards to human motion, social interactions require predicting the timing of others' actions to achieve temporal coordination in joint actions [24]–[25]. Action understanding and interactions would be facilitated if we shared a common, specialized time base with others, a time base rooted in the same mechanisms used for timing our own motor actions and for understanding causality [18], [20]. Conversely, interaction with inanimate things would be improved if the time base of visuomotor coordination was calibrated using internal models of passive dynamics [7], [21].

To determine if time is calibrated differentially as a function of perceived animate or inanimate context, here we used interference paradigms in which a timing task was run concurrently with the presentation of different computer-graphics characters in the background. The timing task served as a probe to reveal potential biases or distortions of time induced by the characters. In separate sessions, the scene displayed characters which could differ in terms of human or artificial appearance and kinematics, while the low-level features of the stimuli were matched as much as possible across conditions. In particular, we used several different types of animate characters with a variable extent of naturalness. The most natural character type was denoted as *Biological-Motion* because it was endowed with the kinematics recorded from a real human actor [19]. Naturalness and animacy were degraded in the character type denoted as *Upside-Down* where the human figure was displayed in an inverted orientation, and in the character type denoted as *Time-Shifted* whose motion was perturbed. These animate characters were contrasted with inanimate characters (*Rigid-Translation*, *Double-Pendulum*, *Whirligig*) whose appearance and/or motion were clearly artificial. Crucially, default trials always depicting the same static character of a standing person were randomly intermingled with the dynamic trials in both animate and inanimate sessions, so as to assess persistent influences of the animate and inanimate contexts.

In two different experimental series, we used either a motor interception task or a perceptual time discrimination task as a probe for testing the effects of different scenes on time estimates. For the interception experiments, we chose a coincidence-anticipation task that involves activations of muscle forces timed on the target arrival at destination. This task relies on automatic sensori-motor processes to compute the time-to-contact [7]. For the time discrimination, we chose a task involving perceptual judgements of temporal durations of a stationary flash [6]. Although in line of principle the background scenes were irrelevant for performing both tasks, we found that the time estimates were systematically shorter in an animate context than in an inanimate context, and this was so irrespective of whether the moving character or the default static character was displayed.

## Results

### Timing an interception movement

#### Task design

**Figure 1:**
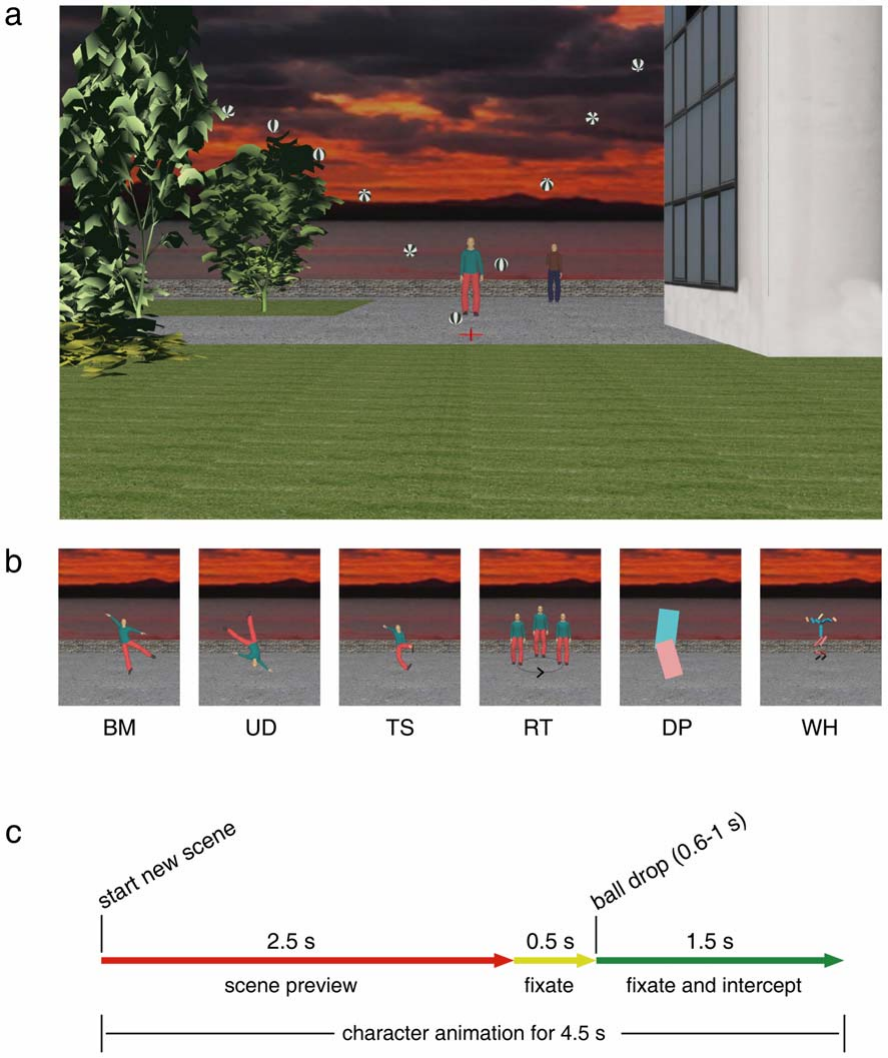
Schematics of the interception experiments. (a) Scene (at 17-m apparent viewing distance) displayed during the *static* trials of the interception experiments. The ball was thrown from the building and hit ground at the centre of the red cross (magnified for clarity in the Figure). Different positions of the ball during its motion are shown for illustrative purposes only. (b) Single frames from the different types of character animation in the *dynamic* trials. Here and in the following figures, BM stands for *Biological-Motion*, UD for *Upside-Down*, TS for *Time-Shifted*, RT for *Rigid-Translation*, DP for *Double-Pendulum*, and WH for *Whirligig*. The RT character is depicted in different positions for clarity, and the motion arrow was not present in the actual movie. (c) Time sequence of events during each trial.

In the first series of experiments, we assessed the effects of viewing different scenes on the fast manual interception of a moving target (Fig. 1a). After a brief preview of the scene, a ball fell under the effect of gravity and bounced away after hitting ground (Fig. 1c). Subjects were asked to press a button when the ball first hit ground. No performance feedback was provided in the main experiments, in order to ascertain the contribution of internal timing mechanisms in the absence of sensory error signals which may correct performance with practice. Initial ball speed was randomized to make descent duration unpredictable from trial to trial. A static or a moving figure was displayed in the near background of the interception point. In the *static* trials of all sessions, the figure depicted a standing human character without implied motion features. Instead, the type of moving figure displayed during the *dynamic* trials varied among sessions, and consisted of either animate or inanimate characters (Fig. 1b). Therefore, the context (animate or inanimate) was defined only by the moving figure of the *dynamic* trials. The context was blocked in separate sessions to avoid carry-over effects from one condition to another one (sessions were run at about 2 weeks distance, and presentation order was counterbalanced across subjects, n = 7).

To assess the tuning of neural time to natural human animacy, we presented six different types of moving characters. 1) A character denoted as *Biological-Motion* provided the reference for natural animacy (Movie S1). This character depicted a human dancer performing a series of smooth steps from classical ballet, moving back and forth around the interception point without jumps. The figure was animated by means of the kinematics accurately recorded from a real human dancer and superimposed upon the facial and bodily forms of the human character. We chose classical ballet to provide a compelling percept of human animacy [26] even under the impoverished conditions of computer graphics. 2) *Upside-Down* (Movie S2): the dancer used in *Biological-Motion* was spatially inverted, taking into account that human actions are more difficult to recognize in upside-down animations [19], [27]. 3) *Time-Shifted* (Movie S3): the motion of each body segment of *Biological-Motion* was randomly time-shifted, independently of one another, while maintaining the anthropomorphic configuration. This manipulation preserved the amplitude and frequency of the original data, but severely perturbed the quality of perceived natural motion. Both *Time-Shifted* and *Upside-Down* served to verify whether unnatural animate motions affect response timing in the same manner as the more natural *Biological-Motion*. 4) *Rigid-Translation* (Movie S4): the standing human figure of the *static* trials was rigidly translated along an elliptic path with simple harmonic motion, resembling the displacement of a picture on a flat surface. *Rigid-Translation* served to verify whether the simple human appearance of the moving figure in an otherwise inanimate context affects response timing in the same manner as *Biological-Motion*. 5) *Double-Pendulum* (Movie S5): two linked mechanical plates freely oscillated back and forth at fundamental frequencies matched to those of *Biological-Motion* (frequencies of the upper and lower link of *Double-Pendulum* were matched with those of the head-trunk and lower limbs of *Biological-Motion*, respectively). The rationale for using this character is that several forms of human movement involve pendular oscillations, and we wanted to assess whether pendular motion per se - irrespective of animacy - affects response timing in the same manner as *Biological-Motion*. 6) *Whirligig* (Movie S6): this character consisted of 14 disconnected rods, whose length, colour, and motion (up to the third harmonic) matched those of the corresponding body segments of the *Biological-Motion* dancer. Although several low-level features of *Whirligig* mimicked those of *Biological-Motion*, its overall appearance and motion looked inanimate.

All six characters were roughly size-matched, and their speed at the point closest to the interception point was comparable (about 1–3°/s, depending on the apparent viewing distance, see *Methods*), much lower than the ball speed at interception (about 20–40°/s). In this manner, the relative temporal contrast [3] between the target (ball speed) and the background (character speed) was comparable across conditions. Similarly, the non-temporal contrast related to the probability of occurrence of the moving character was equalized across conditions (*static* and *dynamic* trials were randomly intermingled in each session). Moreover, the kinematics of all characters, except *Time-Shifted*, complied with the 2/3 power law that relates the instantaneous velocity of a limb end point to the curvature of the geometrical path [28]. This law is typically obeyed by biological motion, as well as by non-biological harmonic motions.

In all *dynamic* trials, the starting frame of the movie was chosen randomly, so that the movie segment displayed simultaneously with the fall of the ball also varied randomly from trial to trial, not to provide spatio-temporal cues for ball interception. The size of the elements in the scene (including the character) was consistent with one of three different apparent viewing distances (*close*, *intermediate*, *distant*), randomized across trials, so as to vary the size of the stimulated visual field and to require the observer to recalibrate the visuo-motor mapping.

Altogether, there were 3 [viewing distance: *close*, *intermediate*, *distant*] x 2 [figure type: *static*, *dynamic*] x 5 [ball descent duration: 0.6, 0.7, 0.8, 0.9, 1.0 s] x 50 [repetitions]  = 1500 trials in each experiment. Instead, the 6 types of moving character (*Biological-Motion*, *Upside-Down*, *Time-Shifted*, *Rigid-Translation*, *Double-Pendulum*, *Whirligig*) displayed during the *dynamic* trials were varied between sessions.

#### Response gradient as a function of character type

**Figure 2:**
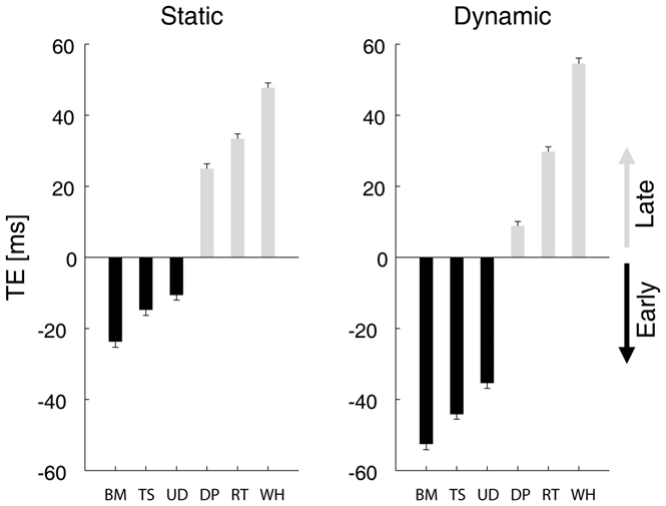
Interception timing error (TE) as a function of character type. Ensemble average TE (± s.e.m.) was computed for all *static* and *dynamic* trials of all sessions involving the six characters of Fig. 1b. TE was computed as the difference between the button-press response time and the duration of ball descent. Negative (positive) values correspond to early (late) responses.

Figure 2 compares the average timing errors (TE) of the interception responses obtained for each separate session involving different character types in the *dynamic* trials. Statistical analysis carried out on the timing errors, pooled over all subjects after averaging over all repetitions of each condition (four-way ANOVA, 3 [viewing distance] x 5 [ball descent duration] x 2 [figure type] x 6 [character type]), showed highly significant effects of character type (*F*_5,1168_ = 113.188, *P*<10^−7^) and figure type (*static* versus *dynamic*, *F*_1,1168_ = 25.180, *P*<10^−6^), less significant effects of viewing distance (*F*_2,1168_ = 3.602, *P*<0.05) and of the interaction between character type and figure type (*F*_5,1168_ = 2.569, *P*<0.05). The effects of the other factors and interactions were not significant.

In contrast with the mean value of the timing errors, the variance across trials did not show any systematic trend as a function of experimental condition (Bartlett's *χ* ^2^ test, *P*>0.18 in all but one subject, in whom the interaction between character type and figure type was significant, *P*<0.005).

Importantly, there was a gradient of the response timing as a function of the character type for both *static* and *dynamic* trials: on average, the earliest (most negative) responses were associated with the *Biological-Motion* condition, while progressively later values were associated with *Time-Shifted*, *Upside-Down*, *Double-Pendulum*, *Rigid-Translation* and *Whirligig* conditions in this order (see Fig. 2). This trend was confirmed by statistically comparing the mean timing errors between different pairs of conditions. The post-hoc Bonferroni *t*-tests were significant (*P*<0.05) in all subjects with the following exceptions: the (between-sessions) comparison between *Time-Shifted* and *Upside-Down* was not significant in two subjects, and the (between-sessions) comparison between *Rigid-Translation* and *Whirligig* was not significant in one subject. It can be noticed that early interception responses occurred with all the characters endowed with some degree of animacy (*Biological-Motion*, *Time-Shifted* and *Upside-Down*), although the negative bias was larger with the more natural animate character (*Biological-Motion*) than with the unnatural characters (*Time-Shifted* and *Upside-Down*).

The graded changes of response timing across the six different types of characters suggest that the neural time estimates required by interception are affected by animacy.

#### Effects in static versus dynamic trials

**Figure 3:**
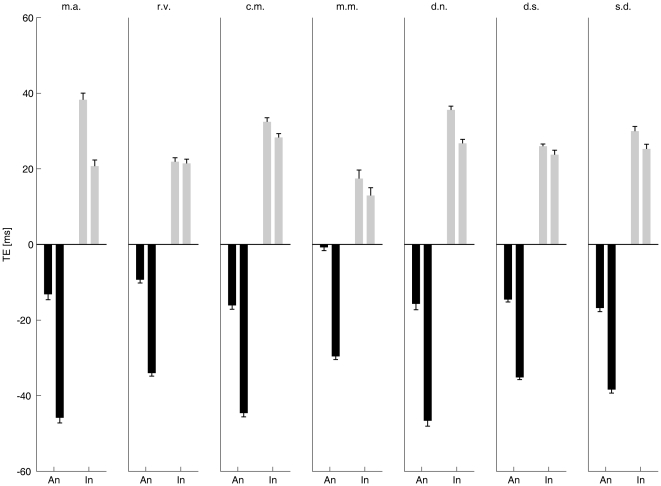
Interception timing error (TE) in individual subjects. Mean TE (± s.e.m.) was computed over all animate characters (*Biological-Motion*, *Upside-Down* and *Time-Shifted*) and over all inanimate characters (*Rigid-Translation*, *Double-Pendulum* and *Whirligig*), and is plotted as An (black) and In (gray), respectively. Left and right bars in each panel correspond to the data for *static* and *dynamic* trials, respectively.

Strikingly, the context (animate or inanimate) biased the interception timing in the same direction in the *dynamic* and *static* trials, although in the *static* trials the visual scene was identical in all sessions and there were no dynamic signals other than those due to ball motion. The subjects who performed all the experiments showed the same trend (the individual responses averaged across all animate characters or all inanimate characters are plotted in Fig. 3). Thirteen additional subjects performed shorter experimental series involving a subset of the six conditions (including at least one animate and one inanimate character). In all but one of these subjects, we found that the mean response timing for animate characters was significantly earlier than the mean value for inanimate characters in both *static* and *dynamic* trials (*t*-test, *P*<10^−3^). In one subject, there was no significant difference (*P*>0.56).

**Figure 4:**
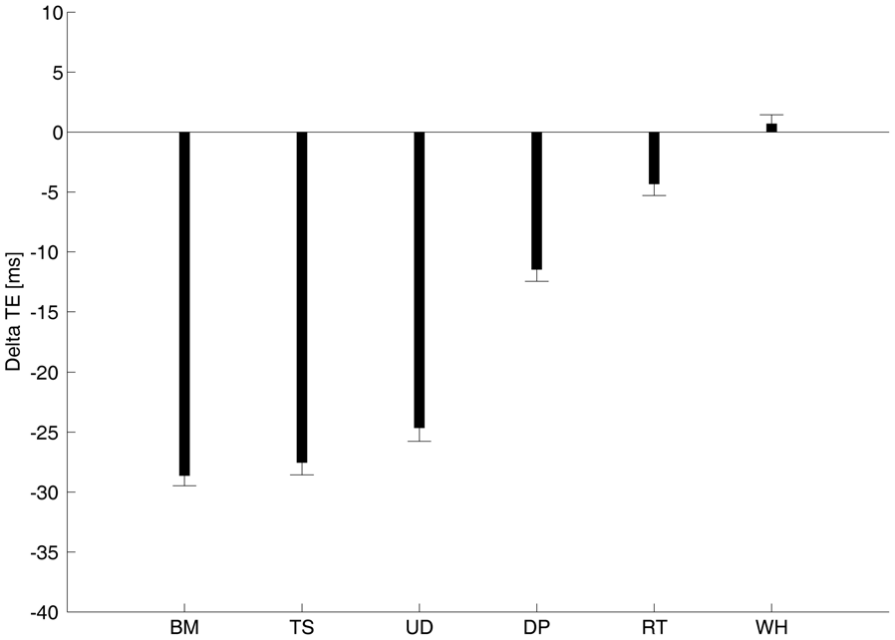
Average (± s.e.m.) difference between the mean timing error in *dynamic* trials and the corresponding value in *static* trials (Delta TE). Delta TE was computed over the 7 subjects who performed all 6 experimental sessions.

The relative size of the time bias in *static* versus *dynamic* trials was not constant across characters, but depended on the specific moving character displayed in the *dynamic* trials of the same session (as also implied by the significant interaction between the character type and the figure type, see ANOVA above). Figure 4 plots the average difference between the value of the mean timing error in *dynamic* trials and the corresponding value in *static* trials. The trend was very similar to that previously described for the timing errors of *static* and *dynamic* trials considered separately (see Fig. 2): the largest absolute difference between *dynamic* and *static* values (corresponding to a more negative timing in the former than in the latter case) was associated with the *Biological-Motion* condition, while progressively smaller values were associated with *Time-Shifted*, *Upside-Down*, *Double-Pendulum*, *Rigid-Translation* and *Whirligig* conditions in this order. All comparisons were statistically significant (post-hoc Bonferroni *t*-tests, *P*<0.05) with the exception of the comparison between *Biological-Motion* and *Time-Shifted*.

#### History of context effects

**Figure 5:**
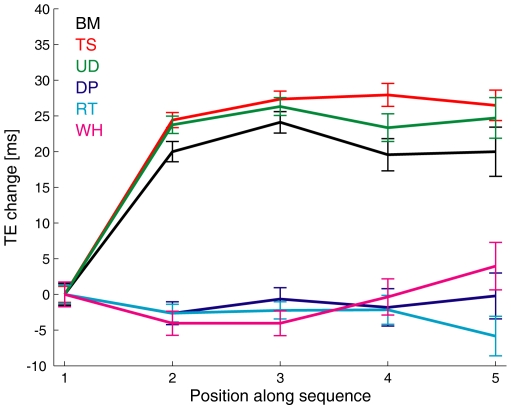
Response adaptation in consecutive *static* trials. Sequences of 3 or more consecutive *static* trials were extracted from all experiments and subjects. The change of timing error in each consecutive trial of the sequence relative to the first trial is graphed as a function of the serial position *i* of the corresponding trial (n = 995 trials for *i* = 1–3, n = 396 for *i* = 4, n = 177 for *i* = 5). The first trial of the sequence was preceded by one or more *dynamic* trial. Notice that the consecutive *static* trials were not identical, because either ball descent duration or apparent viewing distance varied between any two consecutive trials due to the randomization procedure.

So far, we concentrated on the responses averaged over all repetitions of the stimuli. If we consider the history of the interception responses in the course of an experiment, we find evidence of transient after-effects of the moving characters. Figure 5 plots the change of response timing in a sequence of consecutive *static* trials following a *dynamic* trial. The trends were clearly different between the animate sessions considered together (*Biological-Motion*, *Time-Shifted* and *Upside-Down*) and the inanimate ones (*Rigid-Translation*, *Double-Pendulum* and *Whirligig*). In the inanimate sessions, there was no significant change of timing in 5 consecutive *static* presentations (one-way ANOVA on timing errors, all *P*>0.29), indicating that interception timing was affected by this context steadily, independently of transient after-effects. By contrast, in the animate conditions, the timing error depended significantly on the serial position of the trial in the sequence (*P*<10^−5^). On average, response timing was significantly earlier in the first trial than in the following trials of the sequence (post-hoc Bonferroni *t*-tests, *P*<0.05), while there was no significant difference between the latter ones. In other words, the effects of an animate context were stronger in the *static* trials immediately following the presentation of the moving character than in the subsequent *static* trials. However, although attenuated, also the time bias associated with the animate context persisted over at least 5 consecutive *static* trials (corresponding to ∼22 s of continuous presentation of the still character). Indeed, when we considered only the subset of *static* trials preceded by another *static* trial, the mean timing error of the animate sessions was still significantly smaller than the mean timing error of the inanimate sessions (paired *t*-test, *P*<0.05). Moreover, gradients of response timing as a function of character type qualitatively very similar to those of Fig. 2 and Fig. 4 were also obtained using only the subset of *static* trials preceded by another *static* trial. No significant trends were observed in the analogous sequences of 5 consecutive *dynamic* trials, either in the animate or the inanimate sessions (one-way ANOVA on timing error values, all *P*>0.33).

Apart from the described transient changes, we found no significant attenuation of the time biases over the whole experimental session in either animate or inanimate sessions. This was shown by comparing the mean timing error over the first 10 repetitions starting from session onset with the mean timing error over the last 10 repetitions (two-tailed *t*-test, *P*>0.31), and by performing a linear regression of timing error as a function of all repetitions (*P*>0.23).

#### Control experiments

We verified the long-term stability of the results in 3 subjects by repeating both *Biological-Motion* and *Whirligig* sessions at several (>6) weeks of distance from the original one. In such repeated sessions, the mean response timing for *Biological-Motion* was significantly earlier than that for *Whirligig* in both *static* and *dynamic* trials (*t*-test, *P*<10^−5^), while the corresponding values for each condition *Biological-Motion* or *Whirligig* did not differ significantly between the two homologous sessions (all *P*>0.14).

Additional control experiments were carried out to investigate the specificity of the context effects. One experiment (n = 2) included *static* trials only. Here we found that, in the absence of contextual cues provided by *dynamic* trials, the mean response timing did not differ significantly (*P*>0.25) from the ideal value of zero error. Nor did it differ significantly (*P*>0.15) in a separate *Biological-Motion* experiment in which subjects (n = 3) were given performance feedback in both *static* and *dynamic* trials (see *Methods*), showing that the feedback could overcome the interference exerted by the background character.

### Animacy rating

**Figure 6:**
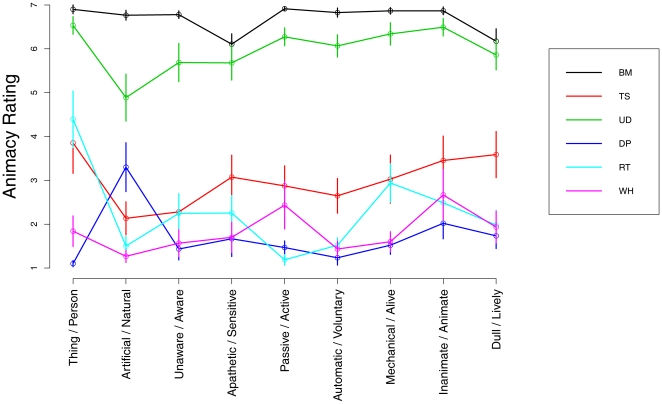
Mean (± s.e.m.) animacy rating computed across all subjects. Ratings for different characters are color-coded (see right inset), and the values for each of the 9 different semantic pairs are plotted in different columns (bi-polar words on the abscissa). Ratings could vary between 1 and 7, higher ratings denoting greater animacy.

In separate experiments, we asked subjects (n = 10) to rate the perceived animacy of the six moving characters (Fig. 1b) projected on the background scene of the interception experiments (but there was no falling ball). Here, the characters were randomly intermingled across trials, and no *static* trials were included. After viewing the movie, subjects rated it on a 7-points scale according to a semantic item drawn randomly from a 9-items questionnaire, higher ratings denoting greater animacy (Fig. 6). Altogether, there were 6 [characters: *Biological-Motion*, *Upside-Down*, *Time-Shifted*, *Rigid-Translation*, *Double-Pendulum*, *Whirligig*] x 9 [questions: *Thing/Person*, *Artificial/Natural*, *Unaware/Aware*, *Apathetic/Sensitive*, *Passive/Active*, *Automatic/Voluntary*, *Mechanical/Alive*, *Inanimate/Animate*, *Dull/Lively*] x 3 [repetitions]  = 162 trials in each experiment.

Statistical analysis carried out on the rating responses, pooled over all subjects after averaging over all repetitions of each condition (two-way repeated measures ANOVA, 6 [characters] x 9 [questions]), showed highly significant effects of both the characters (*F*_5,45_ = 80.131, *P*<10^−6^) and the questions (*F*_8,72_ = 6.761, *P*<10^−5^), as well as a significant interaction between characters and questions (*F*_40,360_ = 5.962, *P*<10^−6^). The average ratings across all questions were: 6.69±0.12 for *Biological-Motion*, 5.98±0.31 for *Upside-Down*, 2.99±0.48 for *Time-Shifted*, 2.28±0.35 for *Rigid-Translation*, 1.83±0.33 for *Whirligig*, and 1.72±0.27 for *Double-Pendulum*. By performing a linear regression of the response timing of the interception experiments for the six characters versus the average animacy rating of the corresponding characters, we found a significant correlation (R^2^ = 0.665, *P*<0.05), in agreement with the hypothesis that the time bias of interception is related to the perceived animacy of the moving characters. Notice, however, that the characters were ranked for perceived animacy in a slightly different order relative to the order found for interception timing.

### Time discrimination

**Figure 7:**
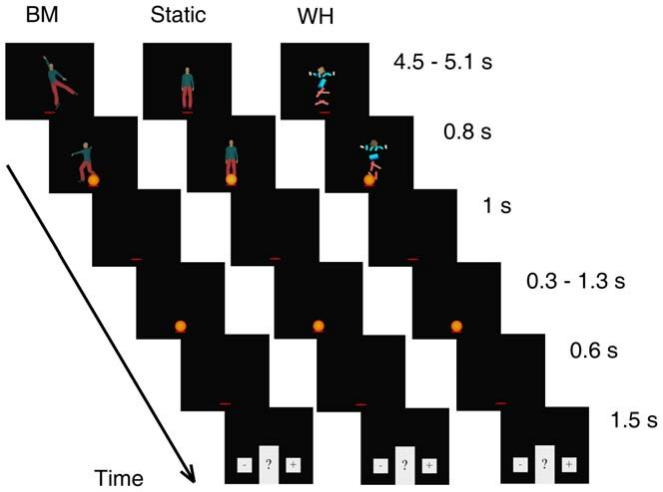
Schematic of the binary choice experiments on duration judgements.

In the next series of experiments, we sought to extend our observations to explicit judgements of perceived time duration of a flash. Our aim was to verify whether the apparent duration of a standard stimulus is affected by the preview of an animate or inanimate movie. We presented subjects (n = 5) with a central fixation cross and a movie of variable duration that involved a static or a moving figure, randomized on a trial-by-trial basis (Fig. 7). The moving figure consisted of the *Biological-Motion* dancer or the *Whirligig* object in animate and inanimate sessions, respectively (order counterbalanced across subjects). We chose these two characters from the full set because they had yielded highly contrasted results in the previous experiments involving either the interception of the moving target or animacy rating. In both sets of sessions, the static figure consisted of the standing human character, as in the interception experiments. The standard stimulus (a stationary sphere) was lit for a fixed duration during the final segment of each movie. Then the screen blanked (except for the fixation cross), and the comparison stimulus was lit for a variable duration. Subjects were asked to indicate whether the comparison was longer or shorter in duration than the standard. The starting frame of the movie in the *dynamic* trials was chosen randomly, so that the movie segment displayed simultaneously with the display of the standard flash also varied randomly from trial to trial.

**Figure 8:**
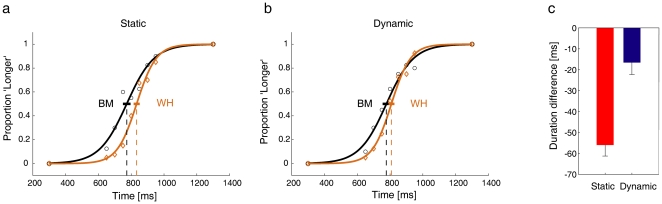
Perceived duration of the standard flash in an animate or inanimate context. (a–b) Psychometric functions for subject P.C., *static* (a) and *dynamic* (b) trials. The graphs show the proportion of times the comparison stimulus appeared to last longer than the standard (360 trials in each panel, 40 repetitions for each of the 9 comparison durations). Data from *Biological-Motion* and *Whirligig* sessions are plotted with black and brown symbols, respectively. The vertical lines (placed on the 50% point of the psychometric functions) denote the mean PSEs of the different conditions, and the horizontal error bars denote the 95% confidence intervals of mean PSE. (c) Average values over all subjects of the difference between the PSE for *Biological-Motion* and that for *Whirligig* (vertical error bars show the s.e.m.). Negative values indicate that the PSE of *Biological-Motion* was shorter than that of *Whirligig*, for both *static* (red) and *dynamic* (blue) trials.

The point of subjective equivalence (PSE) at 50% of the psychometric function estimates the perceived duration of the standard stimulus. This stimulus was perceived as having a systematically shorter duration when it was presented in *Biological-Motion* sessions than when it was presented in *Whirligig* sessions, during both *static* and *dynamic* trials. The results from a representative subject are plotted in Fig. 8a–b, and summary results from all subjects are plotted in Fig. 8c. In Fig. 8a–b, the PSE was 773±23 ms (95% confidence interval, n = 360), 777±22 ms, 833±18 ms, and 808±17 ms for *static Biological-Motion*, *dynamic Biological-Motion*, *static Whirligig*, and *dynamic Whirligig*, respectively. Average values over all subjects of the difference between the PSE for *Biological-Motion* and that for *Whirligig* are plotted in Fig. 8c (both values significantly different from 0, two-tailed *t*-test *P*<0.005).

Although the temporal frequencies of the *Whirligig* motion largely matched those of the *Biological-Motion* dancer, it could be that other unmatched cues (e.g. shape) from these two moving figures distorted time perception differently in *Biological-Motion* and *Whirligig* dynamic trials. On the other hand, in the *static* trials the effects on time perception were even stronger than those in the *dynamic* trials (see Fig. 8), although the visual scene of the *static* trials was identical in *Biological-Motion* and *Whirligig* sessions, and there were no dynamic signals in the background.

In contrast with the PSE, the slope of the psychometric function (which indicates the sensitivity of the temporal judgment) did not depend systematically on the *Biological-Motion* or *Whirligig* condition. The slope was lower in *Biological-Motion* than in *Whirligig* in two subjects (including the subject of Fig. 8a–b), whereas the opposite was true in 3 other subjects. On average, the slope did not differ significantly (*P*>0.4) across conditions. The overall mean Just Noticeable Difference (JND, inverse of the slope) was 73±24 ms (mean ± SD) across all conditions, falling in the range of values previously reported for the discrimination of flash durations comparable to that of our stimuli [2].

## Discussion

We showed that two very different kinds of time estimates were similarly affected by animacy. Without performance feedback, subjects rushed to intercept a moving target in an animate context, whereas they dragged in an inanimate context. Also, subjects estimated the duration of a stationary flash as being shorter in an animate than in an inanimate context. These results suggest that, in both an automatic form of motor timing and a cognitive form of time perception, the observers became tuned to a time base intrinsically linked to a background character, and that such time base differed as a function of the relative animacy.

It is unlikely that the differential effects of animate and inanimate characters on time estimates resulted from different low-level features of the visual stimuli. The spatial position and size of all tested characters was comparable, as was their speed and temporal frequency. Moreover, the kinematics of all characters, except the *Time-Shifted* character, complied with the 2/3 power law typical of harmonic motion, biological or non-biological [28]. Local adaptation of the visual field by the moving stimuli cannot account for the present results. In this regards, it has previously been shown [8] that local adaptation is induced by fast-moving stimuli (20 Hz) but not by slow-moving stimuli (5 Hz). This effect is selective for the temporal frequency of the adapting stimulus. In contrast, all our background moving stimuli were slow, while the speed of the falling ball was more than an order of magnitude faster than the speed of the moving character. Moreover, local adaptation of the visual field reduces the perceived duration for stimuli presented to the position of the adapting stimuli [8], whereas we observed an increase of the perceived duration with the inanimate characters.

Exposure to a moving pattern may reduce the perceived speed of subsequent moving patterns [11]. Thus one might argue that background motion (in dynamic trials of the interception experiments) affected the estimates of ball speed around the time of central trigger of the motor response (about 200 ms before button-press, see 7). Furthermore one might expect that different types of background motion may induce different distortions in the estimates of ball speed, thus resulting in different timing errors in each experimental condition. However, several observations argue against this possibility. First, there was no significant interaction between the ball descent duration (or equivalently ball speed) and the presence/absence of background motion (factor figure type). Second, the velocity of background motion was about an order of magnitude slower than that of the ball at 200 ms before landing, and background velocity was roughly comparable across conditions. Critically, qualitatively similar effects on time estimates were observed irrespective of whether a moving character or a default static figure was displayed in the scene. Therefore, it appears that the intermittent presence of a given moving character in the background was sufficient to determine a specific, persistent bias in the time estimates that carried over to the static background, at least to some extent.

It could be argued that the moving characters acted as distractors depriving the timing tasks of attentional resources, with the most salient characters leading to the greatest task interference [29]. In general, salient distractors delay interception and reaction time responses [29], [30], while they compress perceived time in discrimination tasks [28], [31]. However, the time biases we found are inconsistent with these attentional effects. In fact, *Biological-Motion* and *Whirligig* induced time biases of a comparable absolute magnitude and variability, but in opposite directions: *Biological-Motion* compressed perceived time and anticipated the interception responses, whereas *Whirligig* expanded perceived time and delayed interception. Moreover, there was no significant attenuation of the time biases with repeated presentations of the moving character, contrary to the notion that pairing a timing task with a concurrent well-practiced distractor leads to a systematic reduction of the time bias with repeated presentations [28].

Although general arousal mechanisms cannot easily explain the present findings, specialized attention systems which can differentially detect animate and inanimate targets in complex scenes [32] may well be engaged by our moving characters. Domain-specific subsystems within visual attention mechanisms appear well suited to monitor separately the timing of human or inanimate motion. Domain-specific time tuning is supported by the finding that the human *Biological-Motion* character biased interception timing in the opposite direction relative to the inanimate characters *Double-Pendulum*, *Rigid-Translation* and *Whirligig*. Furthermore, a decrease of natural animacy in the human dancer - from *Biological-Motion* to *Upside-Down* and *Time-Shifted* - diminished the interference on response timing, consistent with the interpretation that the effects reflect a neural tuning to natural human animacy.

One may wonder why the unnatural *Upside-Down* and *Time-Shifted* affected the time responses at all. In particular, *Time-Shifted* disrupted the series of recognizable ballet steps and violated the 2/3 power law typical of harmonic motion, eliminating the impression of a dance along with its implicit musical *tempo*. However, the tuning of neural time to human animacy may be relatively broad, and may not necessarily depend on the absolute compliance of the observed motion with the 2/3 power law. Furthermore, several elemental visual properties may contribute toward a sense of human animacy. A current view is that perceived biological motion may depend on a two-stage processing: an early bottom-up stage where local motion signals are integrated to reconstruct individual body segments (arms, legs etc), and a subsequent top-down stage where individual segments are combined to represent whole agents [33]. It has also been argued that the mechanisms responsible for processing local biological motion signals retrieve the agent's motion direction, but also aid in assessing the animate nature of the agent [34]. Accordingly, we conjecture that elemental biological components were detected not only in *Biological-Motion* but also in *Upside-Down* and *Time-Shifted* by local motion processing, and this detection resulted in a partial entrainment of a “human animate” time base.

On the other hand, the simple human appearance of the *Rigid-Translation* character was not sufficient to entrain the “human animate” time base, presumably because its motion was entirely artificial, resembling the displacement of an inanimate picture. Indeed, *Rigid-Translation* affected the interception responses in the same direction as the inanimate characters *Double-Pendulum* and *Whirligig*. Notice that *Double-Pendulum* delayed the responses substantially less than *Rigid-Translation* and *Whirligig*, possibly because the kinetics of *Double-Pendulum*, but not that of *Rigid-Translation* or *Whirligig*, was congruent with the kinetics of the ball to be intercepted (gravity was the only force acting on both *Double-Pendulum* and ball motion).

A role of kinetics in shaping time estimates has previously been suggested [1], [7], and is consistent with the idea that also the animate-inanimate distinction hinges on expected kinetic differences: internal versus external energy sources are generally assumed for animate versus inanimate motion, respectively [18], [35]. By design, our animate characters (*Biological-Motion*, *Upside-Down*, and *Time-Shifted*) were endowed with simulated internal energy sources (derived from the original dancer whose motion had been captured), whereas the inanimate characters (*Rigid-Translation*, *Double-Pendulum* and *Whirligig*) were driven by simulated external energy sources only. Expected kinetics may also account for the finding that time appeared to run faster in an animate context than in inanimate one, at least in our experimental conditions. Indeed, in the ancestral world where action monitoring presumably evolved, animate targets tend to move more frequently than inanimate targets, and their behaviour is more time-sensitive. Accordingly, changes in animate targets are detected faster than those in inanimate targets [32].

The most remarkable finding of the present experiments was that the time estimates were systematically affected by the animate or inanimate context even during *static* trials, several seconds after the offset of moving characters. The specific, persistent bias in the time estimates is indicative of a contextual priming on the observers' ability to represent elapsed time. One may speculate that animate context conveyed “animacy” also to the standing human figure of the *static* trials as if the observers expected that this figure would start moving at any moment. Instead, the same figure perhaps borrowed the passive features of the inanimate characters in the corresponding context.

The relative size of the time bias in *static* versus *dynamic* trials was not constant, but varied as a function of the experiment type (interception or time discrimination) and character type. In the time discrimination experiments, the bias was stronger in *static* than in *dynamic* trials, possibly because in the latter trials the moving characters may have affected both the standard and the comparison stimuli, reducing the measurable effects, whereas no such direct effect of motion could occur in the *static* trials. In the interception experiments, instead, we found a greater anticipation of the timed responses in *dynamic* trials than in *static* trials with animate characters (Fig. 4). Significantly, the difference between *dynamic* and *static* values followed the same trend as did response timing: the largest absolute difference was associated with the *Biological-Motion* condition, while progressively smaller values were associated with *Time-Shifted*, *Upside-Down*, *Double-Pendulum*, *Rigid-Translation* and *Whirligig* conditions in this order. Moreover, transient after-effects of the moving characters onto the immediately following *static* trial were observed in the animate sessions exclusively (Fig. 5). These results suggest that, on top of overall context effects, dynamic on-line signals from the moving characters could play an important role in affecting the interception timing, but that this role was related to the specific character: the greater was its natural animacy, the larger the time modulation by dynamic signals.

The bulk of our results suggests that neural time mechanisms involve systems differentially tuned to animate and inanimate motion. At a basic level, visual motion processing requires to set the observed events in sequence and to compare their spatial locations over time intervals: in other words, it requires filters oriented in space-time. Specialized spatio-temporal filters probably perform better than general-purpose filters [8], [9]. At a higher level, specialized time calibration may be important for decoding functional aspects of dynamic events, such as the significance of specific actions in biological motion or the fate of object motion [24].

Specialization of the neural time estimates would enhance the temporal resolution of visual processing and the ability to predict critically timed events. Several previous observations might be reconciled within our proposed framework that vision of human and inanimate motions may exert differential top-down influences on automatic processes computing time. Thus, it is known that vision of upright point-light human movement enhances the detection of coherence of local dot motion above the level attained during vision of upside-down movement [36]. Vision of upright human movement also enhances the detection of rolling motion [37], and suppresses perceptual asynchronies in detecting motion onset and colour/form onset [38]. Moreover, animacy increases the discrimination of walking direction in point-light figures [34]. The hypothesis of specialized time calibration for human movement may explain why people are so accurate at predicting the timing of others' actions [25]. Interestingly, visual discrimination of point-light motion of two interacting agents is worse when the two actions are desynchronized [24]. Neri et al. argued that time-locking in a behaviourally meaningful way between interacting agents provides an implicit temporal cue and the additional agent can be used to predict the expected trajectory of the relevant agent with better precision. On the other hand, observation of movements of others may interfere with our own actions when observed and performed actions are dissimilar [39], [40]. Moreover, artificially speeding up (slowing down) point-light animations of human movement determines faster (slower) reaction time responses [41], and duration judgments are compressed during slow-motion video sequences of natural biological motion [4]. All these effects typically weaken or disappear altogether when the animacy perception is degraded.

Here we have been able to experimentally dissect the effects of seeing animate motion from those of seeing inanimate motion by eliminating performance feedback. The large errors in the time estimates we observed under these conditions, however, do not contradict our hypothesis that specialized time calibration enhances the temporal resolution of visual processing and the ability to predict critically timed events, nor do they imply that the brain is unable to deal with the two motion categories (animate and inanimate) at the same time, as is often required in real life. In fact, in separate experiments we showed that performance feedback completely overcame the interference of the *Biological-Motion* character on interception and led to accurate responses. This is consistent with the hypothesis that time is calibrated by comparing the predictions of a neural model about target kinematics with sensory feedback [4].

Several brain regions presumably participate in encoding time, such as the cerebellum, basal ganglia, frontal and parietal cortices (e.g. [2], [5], [42]). Direct neural correlates of elapsed time in the subsecond range have been found in posterior parietal cortex of the monkey [43], [44]. These regions contain neurons with ramping activities whose slope tightly correlates with the perceived duration in a time discrimination task [43] or with motor response timing in an interception task [44]. The slope of such ramps is probably shaped by spatio-temporal integration of excitatory and inhibitory inputs related to visual-motion, motor intention, and high-order contextual signals. We conjecture that neural attributes of animacy may affect this slope and therefore the internal time estimates. This idea is consistent with the view that neural time corresponds to specific spatio-temporal patterns of activity in ensembles of neurons [1], [2].

The contextual priming we described suggests that time modulation takes place late in the visual analysis, perhaps at high representational levels where different items are already identified in categories. In this regards, it is well established that the neural substrates associated with human motion processing are partly distinct from those associated with inanimate motion processing. Observation of human movement activates neural populations from several inter-connected brain regions, including posterior parts of the inferior (pITS) and superior temporal sulci (pSTS), posterior parietal cortex and frontal premotor cortex [19], [20]. In particular, functional magnetic resonance imaging (fMRI) in man showed that pSTS is more active with upright human motion than with upside-down motion or scrambled motion, the latter preserving local kinematics but destroying the configuration of the human body [45]. Also, pSTS is more active with the scrambled motion than with a rigid translation which preserves the configuration of the human body but destroys biological kinematics [46]. These results are reminiscent of the gradient of time distortions we found in the order of *Biological-Motion*, *Upside-Down*, *Time-Shifted*, and *Rigid-Translation* (see Fig. 3), and support the idea that this gradient may reflect a neural tuning to natural human animacy. Neural correlates of perceived animacy and intentional actions have also been described in pSTS/pSTG [22], [47].

When we see someone moving, our brain may covertly simulate the observed action [20], [48]. A neural correlate of motor simulation or motor resonance was described in premotor and posterior parietal areas of the monkey, where ‘mirror’ neurons respond when the monkey performs or views a specific action [20]. In a human fMRI study, activations in premotor cortex, intraparietal sulcus, superior parietal lobe, and pSTS were found in non-expert subjects, ballet and Capoeira dancers who watched movies of other people performing these two types of dances, but the activity was greater when expert subjects watched their own dance style, consistent with the hypothesis that action observation involves an internal motor simulation of the observed movement [26]. The present data suggest that a motor resonance [20], [49] might be obtained by synchronizing neural time to a time base intrinsically linked to the internal simulation of the observed action.

In conclusion, we provided evidence for an influence of human animacy on time estimates. Visual event timers might be tuned to real targets in external space [9] according to the specific natural features of the stimuli, including their animacy, implicating high-level mechanisms for time modulation. Although we considered the possibility that animacy affects neural time, one may also entertain the complementary view that specialized temporal entrainment contributes to animacy attribution.

## Methods

A total of twenty-nine subjects (15 females and 14 males, 28±7 years old, mean ± SD) participated in the study receiving modest monetary compensation. They were right-handed (as assessed by a short questionnaire based on the Edinburgh scale), had normal or corrected-to-normal vision, and were naïve to the purpose of the experiments. They gave written informed consent to procedures approved by the Institutional Review Board of Fondazione Santa Lucia, in conformity with the Declaration of Helsinki on the use of human subjects in research. They sat in front of a 22″ LCD monitor (ViewSonic, model VG2230wm, 1680×1050 pixels, 60 Hz refresh rate) in a dimly illuminated room with the head restrained by a chin rest. Subject-monitor distance was 0.6 and 0.8 m in interception and time discrimination experiments, respectively. Button press responses were recorded by means of National Instruments, PCI 6601 timer/counter at 10 µs resolution. In a subset of experiments (8 subjects), horizontal eye movements were recorded by means of electro-oculogram (EOG) from surface electrodes placed bi-temporally, after calibration. EOG was amplified, low-pass filtered and sampled at 1 KHz by means of National Instruments NI6254 AD converter. We found that the number of trials in which subjects failed to maintain the required fixation (eye movement amplitude >1°, duration >80 ms) was very low (<1% in a given experiment).

All visual stimuli were programmed in C++ using custom software, and rendered using OpenGL 3D on nVidia GeForce 8800 GTX graphics card. The display surface was 470×295 mm. Visual stimuli were defined in a right-handed reference frame with leftward X-axis and upward Y-axis in the frontal plane, plus in-depth Z-axis. Scene projection was computed using on-axis linear perspective, assuming a viewpoint at [0, 1.2 m, -D] and looking at point [0, 1.2 m, 0]. The fixation point was located at the origin [0, 0, 0] of this frame. D (horizontal distance between the origin and the viewpoint) could take one of 3 different values (17, 22.1, or 28.7 m) in the interception experiments, while it was fixed at 17 m in the time discrimination experiments. The position of the observer relative to the screen was adjusted to keep the viewing angle congruent with the above parameters. Timing of the visual stimuli and motor responses were strictly controlled by linking the duration of stimulus presentation to a counter of screen refreshes. To ensure precise control of timing, all moving stimuli were created using look-up table animations.

### Interception experiments

The scene subtended 43° by 28°, horizontal and vertical visual angles, respectively. In the following, we report the visual angles for the apparent viewing distance of 22.1 m (the values for the other viewing distances can be derived by straightforward trigonometry). The scene always included a red cross (0.7° by 0.3°) centred at the origin and drawn on the ground (in perspective, as the other scene elements), a building, a few trees, a human figure in the far background, and another figure (human or inanimate) in the near background (see Fig. 1a). The far figure (0.5° by 1.9°, placed at a distance in depth of 14 m from the origin) was always still, whereas the near one (at a distance of 8 m from the origin) remained static or moved throughout the trial, static and moving figures being randomized across trials (denoted as *static* and *dynamic* trials, respectively). The static figure was always a standing human (0.9° by 3.3°) displayed with Poser-6. The dynamic figure, instead, varied across sessions (see Fig. 1b).

In *Biological-Motion* sessions, a male dancer moved smoothly back and forth around the position which was occupied by the static figure in the *static* trials, without ever leaving ground. The full 10-s movie included several steps from classical ballet, such as *pirouette en dehors à la seconde* and *arabesque*. The dancer's posture never resembled that of the static human figure. This sequence of steps was not gender-specific, as it is typically performed by both males and females. 3D kinematics of the dancer was recorded by means of Vicon 612 motion capture system. The system has 9 cameras, each of which is capable of recording at 100 Hz with images of 1.3 MegaPixel resolution. The dancer wore 62 markers placed on external body references. A model of the dancer's skeleton was composed of 18 segments with a total of 57 degrees of freedom (3 translation dof and 3 orientation dof for the pelvis root segment, plus 3 orientation dof for 17 additional segments hierarchically connected to the root). Fundamental frequencies of motion (computed by Fast Fourier Transform) were 0.1–0.2 Hz for translation, and 0.1–0.8 Hz for rotation (depending on the dof). The first 3 harmonics accounted together for >85% of the data variance at each dof. Processed motion was then imported in Poser-6 to generate the 3D polygonal mesh of the animated actor, and exported in the experimental control program to be displayed at 60-Hz. In each trial, a 4.5-s continuous sequence was extracted from the full 10-s movie by randomly selecting the starting frame within the first 5-s segment.

In *Upside-Down* sessions, the same 3D polygonal mesh of the *Biological-Motion* condition was displayed upside-down, by rotating the whole human figure through 180° around the X-axis at the hips. Otherwise, the kinematics of *Upside-Down* was identical to that of *Biological-Motion*. In *Time-Shifted* sessions, the motion waveform at each of the 57 dof was identical to the corresponding original in *Biological-Motion* but randomly time-shifted, independently at each dof. In *Rigid-Translation* sessions, the standing human figure of the static trials was rigidly translated in the XY plane according to the following equations: 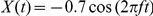, 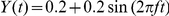 and 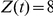, with *f* = 0.3 Hz.

In *Double-Pendulum* sessions, there were two eccentrically linked homogeneous plates. The length and mass of the upper plate matched the total estimated value for the head, trunk and upper limbs of the dancer, whereas the parameters of the lower plate matched those of the pelvis and lower limbs. *Double-Pendulum* motion was computed according to classical mechanics: the two plates were released from non-equilibrium initial configuration and freely oscillated back and forth under gravity around a fixed point placed at the top of the upper plate (negligible friction). Fundamental frequencies of angular motion were 0.56 and 0.58 Hz for the upper and lower plate, respectively.

In *Whirligig* sessions, the figure consisted of 14 close but disjointed rods whose individual length matched that of the corresponding head, trunk and limb segments of the human figure in the other conditions (in *Whirligig* there was no neck, right and left collar, pelvis). Each rod rotated around its centre of mass according to the sum of sinusoids whose amplitude and frequency matched those of the first 3 harmonics (zero-phased) of the angular motion of the corresponding body segment of the original dancer, while all rods translated in 3D according to the sum of the first 3 harmonics of the translational motion of the dancer's pelvis.

On average, the envelope of the character displacement occupied an area 3.7° by 3.5° for *Biological-Motion*, 3.7° by 3.6° for *Time-Shifted*, 3.7° by 3.5° for *Upside-Down*, 3.4° by 3.8° for *Double-Pendulum*, 3.2° by 3.8° for *Rigid-Translation*, and 3.7° by 3.5° for *Whirligig*. The average speed at the character's point closest to the interception point was 0.5 m s^−1^ (1.3° s^−1^) for *Biological-Motion*, 0.7 m s^−1^ (1.8° s^−1^) for *Time-Shifted*, 0.9 m s^−1^ (2.3° s^−1^) for *Upside-Down*, 0.4 m s^−1^ (1.0° s^−1^) for *Double-Pendulum*, 0.9 m s^−1^ (2.3° s^−1^) for *Rigid-Translation*, and 0.6 m s^−1^ (1.5° s^−1^) for *Whirligig*. Compliance of characters' kinematics with the 2/3 power law was verified in the following manner [28]. We first computed the angular velocity *A* and the curvature *C* along the selected trajectory. For *Biological-Motion*, *Upside-Down* and *Time-Shifted* conditions, we selected the trajectory followed by the right wrist of the human actor. For *Double-Pendulum*, we considered the trajectory of the distal plate. We then performed the following linear regression 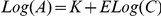



to determine the coefficients *K* and *E*, where *K* is a gain factor depending on the average motion speed and *E* is the power exponent for the relationship between *A* and *C*.

Ideal compliance with the 2/3 power law predicts that 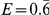. We obtained the following values of *E* for the various conditions: 0.618 for *Biological-Motion* and *Upside-Down*, 0.518 for *Time-Shifted*, 0.669 for *Double-Pendulum*. With regards to *Rigid-Translation* and *Whirligig* conditions, it has previously been shown that harmonic motion (such as that of *Rigid-Translation* and *Whirligig*) satisfies the 2/3 power law exactly [28].

In all experiments, a new scene was shown every 4.5 s. The size of the elements in the scene was consistent with an apparent viewing distance D of 17, 22.1, or 28.7 m, D being randomized across trials. Subjects were free to visually explore the new scene for 2.5 s, then the red cross flickered for 0.5 s indicating that they should fixate at the cross centre for the remaining 1.5 s of the trial (see Fig. 1c). After the flicker period, a textured soccer ball (0.22-m diameter, 0.6°), was thrown downward from an open window of the building and bounced away after hitting ground at the fixation point. The task for the subjects was to press a button with the right index finger when the ball first hit ground, but no performance feedback was provided (except in an additional experiment, see below). Ball trajectory was confined to the vertical plane (Z = 0). The ball fell under gravity (vertical acceleration  = −9.81 m s^−2^), neglecting air drag. Horizontal velocity was kept constant (5 m s^−1^), whereas initial vertical velocity could take one of five different values (−5.39, −3.71, −2.33, −1.14, −0.09 m s^−1^) resulting in five different fall durations (0.6, 0.7, 0.8, 0.9, 1.0 s), randomized across trials. The ball was thrown from a constant height above ground (5 m) but different horizontal positions (X_0_ = −3, −3.5, −4, −4.5, −5 m) to achieve a constant contact point with the ground. The restitution coefficient at the ground was 0.7 (consistent with our measurements performed on a real soccer ball). Ball speed at the interception point was about 11–12 m s^−1^ (28–31° s^−1^).

In separate *Biological-Motion* experiments, performance feedback was provided in each trial. In these experiments, if subjects intercepted the falling ball within an allotted time window (±1 refresh frame relative to that of ball arrival on the ground), the ball exploded blue. If they intercepted too early or too late, the ball was flashed red at the point of incorrect interception.

Overall, 3 different variables were independently randomized trial by trial in each experiment: apparent viewing distance of the scene (3 values), figure type in the near background (*static* or *dynamic*), and ball descent duration (5 values). The exact sequence of trials was different in each session because of the randomization procedure, which only avoided the consecutive repetition of trials with all identical conditions. Therefore, the serial repetition of a given condition was separated from the previous repetition of the same condition by a variable number of trials due to the randomization procedure. Each condition was repeated 50 times, for a total of 1500 trials during each session. Subjects were allowed to pause during the experiment whenever they wanted. Trials with invalid responses (earlier or later than 0.5 s relative to the actual arrival time of the ball on the ground, or no response at all) were rejected and repeated at the end of the experiment (typically there were <1% of such trials per experiment). For each trial we computed the timing error (TE) as the difference between the button-press response time and the fall duration.

### Animacy rating experiments

In each experiment, we presented the same 6 moving characters (*Biological-Motion*, *Upside-Down*, *Time-Shifted*, *Rigid-Translation*, *Double-Pendulum* and *Whirligig*) and the same background scene (at the fixed 17-m viewing distance) as in the interception experiments. Here, the characters were randomly intermingled across trials, and no *static* trials were included. Each trial started with a 4.5-s continuous sequence extracted from the full 10-s movie by randomly selecting the starting frame within the first 5-s segment. Then the character disappeared and a pair of words appeared at the bottom of the scene. The pair was drawn randomly from a questionnaire based on semantic bi-polar items (modified from [50]). The questionnaire included 9 pairs of Italian words whose English equivalent is: Thing/Person, Artificial/Natural, Unaware/Aware, Apathetic/Sensitive, Passive/Active, Automatic/Voluntary, Mechanical/Alive, Inanimate/Animate, Dull/Lively. Subjects were asked to press a numerical key between 1 and 7 to rate the character according to the semantic bi-polar pair (question) currently displayed, higher ratings denoting greater animacy. There was no time limit to deliver the response. After the keypress, a new trial started. Subjects were given 9 practice trials including all questions. During each experiment, each question was randomly presented 3 times for each character, yielding a total of 162 trials (6 characters x 9 questions x 3 repetitions). To check for the internal consistency among the ratings for all 9 questions, we computed the Cronbach's Alpha statistics [51]: the closer to 1 is Alpha, the higher is the internal consistency. Different items of a questionnaire are considered internally consistent if Alpha is above the standard 0.70 cutoff [52]. We found that, on average, Alpha was 0.86 (range 0.72–0.95) showing an acceptable level of homogeneity of the ratings for all 9 questions.

### Time discrimination experiments

Here the scene displayed the red cross against a uniform, black background (see Fig. 7). Subjects were asked to fixate the cross throughout the trial. A character was displayed 4.4° (at the centre of mass) above the cross centre, from trial start until 5.3 to 5.9 s afterwards. The duration of the character display was randomized (16.7-ms discretization) across trials, as was the starting frame of the movie in the *dynamic* trials. During the last 0.8 s of the character presentation, a stationary orange, homogeneous sphere (1°) was displayed with its lower point at the cross centre, providing the standard stimulus for the time discrimination. Then both the character and the sphere disappeared from the screen, and 1-s afterwards the orange sphere re-appeared in the same position for a variable duration (0.3, 0.65, 0.7, 0.75, 0.8, 0.85, 0.9, 0.95, 1.3 s), randomized across trials. The second sphere provided the comparison stimulus for the time discrimination. After a further 0.6-s of blank screen (except for the cross), a question mark appeared sided by a minus and a plus label prompting the subjects to provide the response. They indicated whether the comparison stimulus was longer or shorter in duration than the standard stimulus by pressing a right or left button, respectively. The buttons were mounted at 6-cm distance on a tablet in front of the subjects. The question mark greyed out once the response was acquired. Subjects had 1.5 s to respond, then a new trial started. If they responded before or after the allocated time window, the trial was rejected and repeated at the end of the experiment.

In each experiment, the character displayed at trial start could involve a static (*static* trial) or a moving figure (*dynamic* trial), randomized on a trial-by-trial basis. In separate sessions, the moving figure consisted of the same *Biological-Motion* dancer or *Whirligig* object used in the interception experiments. In all sessions, the static figure consisted of the standing human figure. There were 40 repetitions for each of the 9 durations of the comparison stimulus, and for both the *static* and the *dynamic* conditions, for a total of 720 trials in each session.

Psychometric functions were computed by fitting the logistic function 
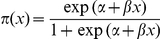
 to the responses. 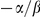 corresponds to the point of 0.5 response probability (i.e. the point of subjective equivalence PSE), and estimates the accuracy of the match. 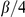 corresponds to the slope of the psychometric function at PSE, and estimates the precision of the match. Data were well fit as shown by a non-significant deviance [53]. Confidence intervals (95%) around the PSE estimates were computed using the delta method [54].

## Supporting Information

Movie S1 **Biological-Motion.** This and the following movies present a low-resolution detail of the actual movie which also included the background scene and the moving ball as in the schematic of Fig. 1a.
(MOV)

Movie S2 **Upside-Down.**
(MOV)

Movie S3 **Time-Shifted.**
(MOV)

Movie S4 **Rigid-Translation.**
(MOV)

Movie S5 **Double-Pendulum.**
(MOV)

Movie S6 **Whirligig.**
(MOV)
